# Supplementary material for: Active site specificity profiling datasets of matrix metalloproteinases (MMPs) 1, 2, 3, 7, 8, 9, 12, 13 and 14
Source: Data Brief. 2016 Feb 22;7:299–310. doi: 10.1016/j.dib.2016.02.036 (PMC4777984; doi:10.1016/j.dib.2016.02.036)
Supplement: Supplementary file 10 — Supplementary material [file mmc10.zip › WebPICS_hMMP9_G_1%/P3prime.html]

 

PICS results


|  |  |
| --- | --- |
| **P3prime\_A**  31 in 146 sites   21.2 %    effects > 10 perc. pnts.   (vice-versa in brackets)  P2prime\_V: 12.1 (18.8) |  |
| **P3prime\_G**  31 in 146 sites   21.2 %    effects > 10 perc. pnts.   (vice-versa in brackets)  P3\_A: 20.4 (28.8)   P2\_K: 12.1 (18.8)   P1\_K: 11.9 (33.3) |  |
| **P3prime\_N**  6 in 146 sites   4.1 %    effects > 10 perc. pnts.   (vice-versa in brackets)  P1\_N: 41.8 (20.9)   P1\_Q: 12.6 (12.6)   P1prime\_V: 25.1 (12.6)   P2prime\_I: 41.1 (19.0) |  |
| **P3prime\_S**  31 in 146 sites   21.2 %    effects > 10 perc. pnts.   (vice-versa in brackets)  P3\_A: -11.9 (-16.7)   P1\_G: 14.6 (21.7)   P1prime\_L: 15.1 (10.6) |  |
